# Supplementary material for: MetaRibo-Seq measures translation in microbiomes
Source: Nat Commun. 2020 Jun 29;11:3268. doi: 10.1038/s41467-020-17081-z (PMC7324362; doi:10.1038/s41467-020-17081-z)
Supplement: Supplementary file 10 — Supplementary Data 7 [file 41467_2020_17081_MOESM10_ESM.zip › File2/Confidence_VeryHigh_Taxonomy/32797_out.krona.html]

Javascript must be enabled to view this page.

members
magnitude
magnitudeUnassigned
count
unassigned
taxon
rank

32797\_out

11

2
superkingdom
11

1239
phylum
11

186801
class
11

order
11
186802

172733

SRS013951\_contig\_number\_27630SRS015960\_contig\_number\_contig-100\_2891.129628SRS019161\_contig\_number\_contig-100\_1779.212158SRS022609\_contig\_number\_contig-100\_6165.141067SRS043701\_contig\_number\_13551SRS049959\_contig\_number\_51672SRS052697\_contig\_number\_41787SRS055017\_contig\_number\_contig-100\_1116.158254SRS077392\_contig\_number\_38360SRS142503\_contig\_number\_contig-100\_1605.342111SRS893383\_contig\_number\_33180
11
species
